# Mediation of the Cardioprotective Effects of Mannitol Discovered, with Refutation of Common Protein Kinases

**DOI:** 10.3390/ijms222212471

**Published:** 2021-11-19

**Authors:** Carolin Torregroza, Chiara O. Glashoerster, Katharina Feige, Martin Stroethoff, Annika Raupach, André Heinen, Markus W. Hollmann, Ragnar Huhn

**Affiliations:** 1 Department of Anesthesiology, Medical Faculty and University Hospital Duesseldorf, Heinrich-Heine-University Duesseldorf, Moorenstr. 5, 40225 Duesseldorf, Germany; Carolin.Torregroza@med.uni-duesseldorf.de (C.T.); Chiara.Glashoerster@hhu.de (C.O.G.); Martin.Stroethoff@med.uni-duesseldorf.de (M.S.); Annika.Raupach@med.uni-duesseldorf.de (A.R.); Ragnar.Huhn@med.uni-duesseldorf.de (R.H.); 2 Institute of Cardiovascular Physiology, Medical Faculty and University Hospital Duesseldorf, Heinrich-Heine-University Duesseldorf, Universitaetsstr. 1, 40225 Duesseldorf, Germany; Andre.Heinen@uni-duesseldorf.de; 3 Department of Anesthesiology, Amsterdam University Medical Center (AUMC), Location AMC, Meiberdreef 9, 1105 AZ Amsterdam, The Netherlands; M.W.Hollmann@amsterdamumc.nl; 4 Department of Anesthesiology, Kerckhoff-Clinic GmbH, Benekestr. 2-8, 61231 Bad Nauheim, Germany

**Keywords:** mannitol, myocardial infarction, preconditioning, adenosine receptor, protein kinase B, protein kinase G

## Abstract

The osmodiuretic agent Mannitol exerts cardioprotection against ischemia and reperfusion (I/R) injury when applied as a pre- and/or postconditioning stimulus. Previously, we demonstrated that these properties are mediated via the activation of mitochondrial ATP-sensitive potassium (mK_ATP_) channels. However, considering Mannitol remains in the extracellular compartment, the question arises as to which receptor and intracellular signaling cascades are involved in myocardial protection by the osmodiuretic substance. Protein kinase B (Akt) and G (PKG), as part of the reperfusion injury salvage kinase (RISK) and/or endothelial nitric oxide (eNOS)/PKG pathway, are two well-investigated intracellular targets conferring myocardial protection upstream of mitochondrial potassium channels. Adenosine receptor subtypes have been shown to trigger different cardioprotective pathways, for example, the reperfusion injury. Further, Mannitol induces an increased activation of the adenosine 1 receptor (A1R) in renal cells conferring its nephroprotective properties. Therefore, we investigated whether (1) Akt and PKG are possible signaling targets involved in Mannitol-induced conditioning upstream of the mK_ATP_ channel and/or whether (2) cardioprotection by Mannitol is mediated via activation of the A1R. All experiments were performed on male Wistar rats in vitro employing the Langendorff isolated heart perfusion technique with infarct size determination as the primary endpoint. To unravel possible protein kinase activation, Mannitol was applied in combination with the Akt (MK2206) or PKG (KT5823) inhibitor. In further groups, an A1R blocker (DPCPX) was given with or without Mannitol. Preconditioning with Mannitol (Man) significantly reduced the infarct size compared to the control group. Co-administration of the A1R blocker DPXPC fully abolished myocardial protection of Mannitol. Interestingly and in contrast to the initial hypothesis, neither administration of the Akt nor the PKG blocker had any impact on the cardioprotective properties of Mannitol-induced preconditioning. These results are quite unexpected and show that the protein kinases Akt and PKG—as possible targets of known protective signaling cascades—are not involved in Mannitol-induced preconditioning. However, the cardioprotective effects of Mannitol are mediated via the A1R.

## 1. Introduction

Ischemia and reperfusion (I/R) injury is defined as the occurrence of myocardial damage due to the restoration of blood and oxygen supply after an ischemic event [1]. A sequence of complex intracellular events, including electrolyte and pH shift as well as the release of proapoptotic factors, is responsible for the incidence of I/R injury and accounts for up to 50% of the final infarcted area [2]. Hence, I/R injury plays a crucial role in patient survival and outcome after myocardial ischemia.

Various pharmacological conditioning strategies have been investigated in recent years, with beneficial results on infarct size and myocardial function after an ischemic event [3,4]. However, these encouraging experimental findings have yet failed to be successfully transferred into clinical trials [5]. One promising cardioprotective substance is the hyperosmolar agent Mannitol, which is routinely used for osmotherapy in the clinical setting [6]. In addition to the treatment of acute intracranial pressure [7], it is also employed in cardiac surgery, showing favorable effects on hemodynamics, coronary blood flow and cardiac function [6]. In addition to known neuro- [8,9,10] and nephroprotection [11], recent research has also demonstrated the cardioprotective properties of Mannitol when applied as a pre- and/or postconditioning stimulus [12]. Besides its hyperosmolar and radical scavenging characteristics [13,14,15,16], we previously showed that mitochondrial ATP-sensitive potassium (mK_ATP_) channels are involved in myocardial protection triggered by Mannitol [12]. However, as Mannitol remains in the extracellular compartment, the question arises as to how the agent confers its intracellular effects. At this point, the Mannitol-induced cardiac signaling pathway upstream of mK_ATP_ channels is unknown.

Two well-investigated intracellular cardioprotective signaling cascades are the reperfusion injury salvage kinase (RISK) [17,18] and endothelial nitric oxide synthase (eNOS)/protein kinase G (PKG) [4] pathways. Both confer myocardial protection by different pharmacological stimuli, ultimately triggering mitochondrial potassium (mK^+^) channels [19,20] and the mitochondrial permeability transition pore (mPTP) [21]. PKG has been demonstrated to modulate both mitochondrial large-conductance calcium-activated potassium (mBK_Ca_) and mK_ATP_ channel openings, while protein kinase B (Akt) is a main target in the RISK pathway triggering mBK_Ca_ channels [22].

G-protein-coupled receptors (GPCRs) are integral membrane proteins expressed by cardiomyocytes [23]. Binding different substances, such as adenosine, activates these respective receptors and converts extracellular stimuli into intracellular signals as part of the above-mentioned cardioprotective pathways [24]. One member of the GPCR superfamily is the adenosine receptor (AR), which mediates several crucial cardiovascular functions, such as heart rate and contraction [25]. Further, different adenosine receptor subtypes have been shown to be involved in various ischemic and pharmacological conditioning strategies [24,26]. The adenosine 1 receptor (A1R) plays an integral role in ischemic and remote ischemic preconditioning, triggering both the RISK and eNOS/PKG pathways [26]. Interestingly, Pingle et al. demonstrated that the administration of Mannitol increases the expression and activation of this specific receptor subtype in renal cells mediating nephroprotection by osmotic diuretics [27]. As the A1R is also found in cardiomyocytes, the question arises as to whether Mannitol might exert its cardioprotective effect via activation of this G-protein-coupled adenosine receptor.

Based on this knowledge, the aim of our study was to analyze whether (1) Akt and PKG are potential intracellular targets involved in cardioprotection by Mannitol and/or whether (2) Mannitol-induced preconditioning leads to the activation of the A1R and the downstream triggering of intracellular cascades.

## 2. Results

### 2.1. Animal Characteristics

There were no differences detected between or within any of the groups in this study regarding wet and dry heart weight and level or time of maximal ischemic contracture (Table 1). The body weight of the animals included in the Man+DPCPX group was significantly lower compared to Man.

### 2.2. Infarct Size 

**Figure 1:**
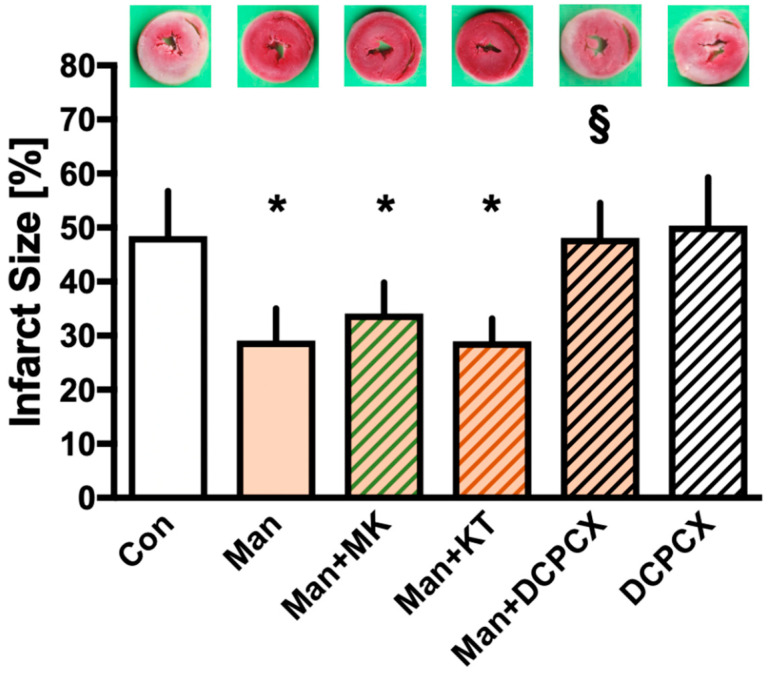
Infarct size measurement. Data are presented as means ± SD, Con = control; Man = Mannitol; MK = MK2206 (Akt inhibitor); KT = KT5823 (PKG inhibitor); DPCPX = adenosine receptor 1 antagonist. * *p* < 0.05 vs. Con, § *p* < 0.05 vs. Man.

Figure 1 presents the results from infarct size determination. Hearts in the control group had an infarct size of 48 ± 8%. Preconditioning with Mannitol led to a significant reduction in final infarct size when compared to the control (Man: 29 ± 6%, *p* < 0.05 vs. Con). Neither the Akt (MK2206) nor PKG (KT5823) blocker had any effect on infarct size reduction by Mannitol-induced preconditioning (Man + MK: 34 ± 6% vs. Man and Man + KT: 29 ± 4% vs. Man, both ns). Co-administration of the A1R inhibitor DPCPX fully abrogated the cardioprotective effects of Mannitol (Man + DPCPX: 48 ± 6%, *p* < 0.05 vs. Man), while the inhibitor itself had no influence on infarct size (DPCPX: 50 ± 9%, ns vs. Con).

### 2.3. Cardiac Function

Comparing hemodynamic data at any measured time point, no differences were detected between the study groups. Regarding coronary flow and left ventricular developed pressure, a significant decrease occurred during reperfusion compared to the baseline within each study group. Hemodynamic variables are shown in Table 2.

## 3. Discussion

The results from this current study demonstrate that Mannitol-induced preconditioning is mediated via the activation of adenosine 1 receptors (A1R); however, PKG and Akt, as two known targets of intracellular signaling cascades, are not involved in myocardial protection by Mannitol. 

In addition to discussing the hyperosmolar and radical scavenging features of Mannitol [15,28,29], we recently further unraveled the underlying mechanisms of cardioprotective properties, demonstrating the activation of mK_ATP_ channels by Mannitol-induced pre- and/or postconditioning [12]. The findings from our previous study showed that the co-administration of the mK_ATP_ channel blocker 5-hydroxydecanoate completely abolished the infarct size-reducing effects of Mannitol [12]. While these results present a connection between Mannitol and known myocardial targets of cardioprotective cascades—specifically mitochondrial potassium (mK^+^) channels—the intracellular pathway triggered by Mannitol upstream of mK_ATP_ channels remains unknown. 

As shown by extensive research, mK^+^ channels are regulated by different intracellular protein kinases as part of cardioprotective signaling cascades, such as the RISK and eNOS/PKG pathways [30,31,32]. Protein kinase B (Akt) and PKG are both well-investigated in this context and have been shown to trigger the downstream activation of mK^+^ channels [22]. Considering this background of knowledge, we focused on these two protein kinases as possible intracellular targets linking Mannitol to mK_ATP_ channels. The applied concentrations of both protein kinase inhibitors were taken from the literature and have been shown to significantly block the respective kinases [33,34,35,36]. The potent and highly specific PKG inhibitor KT5823 was administered in a concentration of 1 µM, in which the blocker, with an IC_50_ of 60 nM and a K_i_ value of 234 nM, does not affect other protein kinases [36]. MK2206 (15 nM) was applied as a selective inhibitor for all three Akt isoforms [35]. Referring to the literature, no effect on other protein kinases can be found. Each blocker itself, in the respective concentration, has no influence on infarct size reduction, as shown in our previous study employing the Langendorff perfusion technique and the exact same experimental setup [36]. Mannitol was administered in a concentration of 11 mmol/L (converting to 1 g/kg body weight as used in clinical practice), which has previously been shown to confer cardioprotection as a pre- and postconditioning stimulus [12]. 

Interestingly and in contrast to the initial hypothesis, findings from our current study demonstrate that neither Akt nor PKG are directly involved in Mannitol-induced preconditioning. Co-administration of the respective kinase blocker had no influence on infarct size reduction by Mannitol. These results were quite unexpected, considering both Akt and PKG have been described extensively as targets in the main cardioprotective signaling cascades, such as RISK and eNOS/PKG. Moreover, for different pharmacological agents—for example, Ramelteon [36] or Sildenafil [37]—it was shown that these respective kinases trigger intracellular mK^+^ channel activation [4]. Hence, more research is necessary to elucidate the intracellular targets upstream of mK_ATP_ channel activation by Mannitol. One potential enzyme in this context might be protein kinase C (PKC), more specifically isoform epsilon (PKCε) [22,38,39]. The role of PKC in ischemic (IPC) and pharmacological preconditioning has been investigated extensively [22,40]. Hassouna et al. [41] indicate that PKCε is located upstream of mK_ATP_ channels in the IPC-induced signaling cascade. In line with these findings, Pravdic et al. [42] demonstrated the PKCε-dependent inhibition of the mitochondrial permeability transition pore (mPTP) by preconditioning with Isoflurane. Thus, future studies could investigate PKC as an intracellular target of Mannitol-induced preconditioning upstream of mK_ATP_ channel activation. 

In addition to the so far underexplored myocardial signaling cascade, the question remains as to how exactly Mannitol triggers intracellular targets considering the substance remains in the extracellular compartment. Several different GPCRs have been shown to be activated by pharmacological substances triggering cardioprotective pathways, such as RISK and eNOS/PKG [23]. Up to this point, no receptor has been linked to Mannitol-induced myocardial protection. However, Pingle et al. [27] investigated the nephroprotective properties of osmotic diuretics in renal cells demonstrating involvement of the A1R. The results from their study indicate that Mannitol induces an increase in A1R expression in renal proximal tubular cells via nuclear factor kappa B (NF-κB). Further, the osmotic diuretic also activates the A1R, leading to nephroprotection [27]. Adenosine receptor-mediated cardioprotection has been well-investigated in recent years, focusing on different receptor subtypes, species and experimental protocols [24,26]. The activation of the A1R in cardiomyocytes is crucial in conferring cardioprotection by ischemic and remote ischemic preconditioning [43]. Through binding ligands to the A1R, extracellular stimuli are converted into intracellular signals. Various intracellular targets have been linked to adenosine receptor activation, such as the phosphorylation of Akt [44], stimulation of cAMP and protein kinase A pathway [25], increasing levels of inducible nitric oxide synthase (iNOS) [26] and the activation of mK^+^ channels [25]. While early studies focused on the A1R, several subsequent investigations have also reported adenosine 2 and 3 receptor agonists as cardioprotective agents. Based on the above-mentioned information, we tested the hypothesis of whether Mannitol exerts its cardioprotective effects via the activation of the A1R in cardiomyocytes. We employed the radioligand 8-cyclopentyl-1,3-dipropylxanthine (DPCPX), a selective antagonist with high affinity for the A1R [45]. The applied DPCPX concentration of 200 nM was taken from a previous study on Isoflurane-induced cardioprotection in isolated rat hearts at the Langendorff apparatus [46]. A radioligand study by Lasley et al. [47] reported DPCPX Ki-values of 0.45 nM for the A1R in cardiomyocytes with a 700-fold A1-selectivity compared to other adenosine receptor subtypes. Thus, the DPCPX concentration (200 nm) in our study is sufficient for the inhibition of the A1R, while not affecting the A2 and A3 receptor. 

Interestingly, in addition to the above-discussed involvement of PKC in mK_ATP_ channel activation, the protein kinase has also been linked to adenosine receptor activation. Experimental studies on these GPCRs demonstrate that PKC is located downstream of adenosine receptor activation [48], specifically the A1R [49]. Hence, PKC might be a potential connection between A1R activation by Mannitol and the downstream triggering of mK_ATP_ channels, independent of Akt and PKG. However, further research is needed investigating a possible interaction of the A1R, PKCε and mK_ATP_ channel openings in Mannitol-induced preconditioning.

### Limitations

While our results indicate that neither Akt nor PKG are involved in the downstream activation of Mannitol-induced preconditioning, we did not measure protein kinase activity in our experiments. However, all inhibitors employed in this study were used in concentrations specific for the respective protein kinase and have been shown to sufficiently block Akt or PKG in previous studies [36]. Further, we investigated the cardioprotective properties of Mannitol in isolated hearts in vitro, focusing on the heart itself without other influencing factors. In future studies, these beneficial effects should be further investigated in vivo. Up to this point, there is only limited knowledge on the underlying mechanisms of Mannitol-induced preconditioning. While our current results give more insight on receptor activation and possible downstream targets, future studies are needed to elucidate whether other membranous candidates and intracellular enzymes are involved in myocardial signaling by Mannitol. 

## 4. Materials and Methods 

All experiments were performed in accordance with the *Guide for the Care and Use of Laboratory Animals,* published by the U.S. National Institute of Health (NIH publication No. 85-23, revised 1996), after approval by the local Animal Care and Use Committee of the University of Duesseldorf (project number O27/12). 

### 4.1. Surgical Preparation

The present study was performed in vitro on the isolated hearts of 2–3-month-old male Wistar rats employing the Langendorff heart perfusion technique [50]. Prior to decapitation, animals were anesthetized by intraperitoneal injection of pentobarbital (80 mg/kg body weight, Narcoren, Merial, Germany). After the thoracotomy, hearts were excised and placed onto the Langendorff apparatus. Perfusion was established via a pressure- and temperature-controlled (80 mmHg, 37 °C) inflow of Krebs–Henseleit buffer (118 mM NaCl, 4.7 mM KCl, 1.2 mM MgSO_4_, 1.17 mM KH_2_PO_4_, 24.9 mM NaHCO_3_, 2.52 mM CaCl_2_, 11 mM glucose and 1 mM lactate) to the isolated hearts. Hemodynamic measurements were achieved by inserting a saline-filled balloon via the left atrium into the left ventricle with a set end-diastolic pressure of 4–6 mmHg. The analogue-to-digital converter (PowerLab/8SP, ADInstruments Pty Ltd., Castle Hill, Australia) along with the program Labchart 8.0 for Windows (ADInstruments Pty Ltd., Castle Hill, Australia) allowed for the continuous measurement of hemodynamic data, including heart rate, left ventricular end-systolic pressure (LVESP), left ventricular end-diastolic pressure (LVEDP), left ventricular developed pressure (LVDP) (calculated as LVESP—LVEDP) and coronary flow. Upon the completion of each experiment, hearts were frozen overnight and then cut into 8 transverse 2 mm slices per heart, starting at the apex. For the determination of the final global infarct size, all heart slices were stained with 0.75% triphenyltetrazolium chloride (TTC) solution. Finally, a blinded investigator measured the total non-viable infarcted area as a percentage of the left ventricle using planimetry (SigmaScan Pro5 software, Systat Software Inc., San Jose, CA, USA) [33]. 

### 4.2. Experimental Protocol

**Figure 2:**
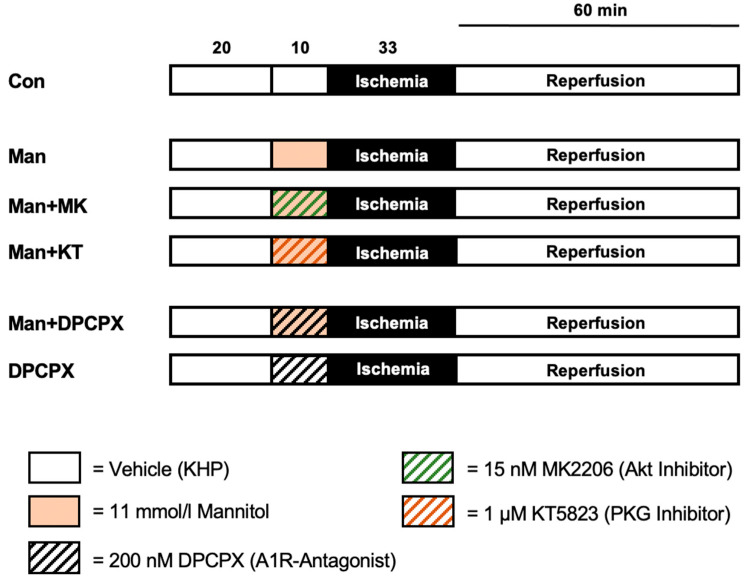
Experimental protocol. Con = control; Man = Mannitol; MK = MK2206 (Akt inhibitor); KT = KT5823 (PKG inhibitor); DPCPX = adenosine receptor 1 antagonist.

Animals were randomly assigned to 6 experimental groups (*n* = 7 per group). The experimental protocol is displayed in Figure 2. After a 20 min adaption period, all hearts received vehicle, Mannitol and/or one of the respective blockers at an infusion rate of 1% coronary flow for 10 min as a preconditioning stimulus. Subsequently, perfusion was stopped, initiating 33 min of global ischemia, followed by 60 min of reperfusion. 

For the treatment with Mannitol (Man), we used a concentration of 11 mmol/L which has previously been shown to induce significant infarct size reduction when applied as a pre- or postconditioning stimulus [12]. In further groups, Mannitol was administered in combination with either the Akt (MK2206, 15 nM) or the PKG (KT5823, 1 µM) blocker. The concentrations of the respective inhibitors employed in this study were taken from the literature [36]. Finally, Mannitol was combined with 200 nM of the A1R blocker DPCPX (Man+DPCPX). To rule out a possible effect of the receptor blocker on the infarct size, it was also given individually (DPCPX). 

Control (Con): Hearts were perfused with Krebs–Henseleit buffer (KHB) as vehicle for 10 min before ischemia.

Man: Mannitol was applied in a concentration of 11 mmol/L for 10 min before ischemia.

Man + MK: 11 mmol/L Mannitol and 15 nM MK2206 were applied simultaneously for 10 min before ischemia. 

Man + KT: 11 mmol/L Mannitol and 1 µM KT5823 were applied simultaneously for 10 min before ischemia. 

Man + DPCPX: 11 mmol/L Mannitol and 200 nM DPCPX were applied simultaneously for 10 min before ischemia. 

DPCPX: Hearts were perfused with 200 nM DPCPX for 10 min before ischemia. 

### 4.3. Statistical Analysis

#### 4.3.1. Sample Size Analysis

Sample size calculation (GraphPad StatMate™, GraphPad Software, San Diego, CA, USA) revealed a group size of *n* = 7 for detecting a 25% mean difference and a standard deviation of 15% in infarct size (power 80%, α < 0.05 (two-tailed)).

#### 4.3.2. Statistical Approach

All data included in this study are presented as mean ± standard deviation (SD). The primary endpoint was infarct size determination, analyzed by a one-way analysis of variance (ANOVA) and a Tukey post hoc test. A two-way ANOVA and a Tukey post hoc test (GraphPad Software V7.01, San Diego, CA, USA) were performed for comparison of the hemodynamic data between groups and within each group at different time points. *p* < 0.05 was considered statistically significant.

## 5. Conclusions

The current investigation demonstrates that Mannitol-induced preconditioning is mediated via the activation of the A1R in cardiomyocytes with the downstream triggering of the mK_ATP_ channel opening. Further, results from our study show that neither Akt nor PKG—as two possible targets of known cardioprotective pathways—are involved in the signaling cascade of myocardial protection by Mannitol. Thus, these findings give a more detailed description of the underlying mechanisms—which were to this point unknown—involved in the cardioprotective properties of Mannitol.

## Figures and Tables

**Table 1:** Weights and ischemic contracture.

	*n*	Body Weight (g)	Heart Weight Dry (g)	Heart Weight Wet (g)	Time of Max. Ischemic Contracture (min)	Level of Max. Ischemic Contracture (mmHg)
Con	7	304 ± 29	0.17 ± 0.02	1.13 ± 0.08	15 ± 1	66 ± 14
Man	7	313 ± 28	0.17 ± 0.02	1.10 ± 0.08	16 ± 2	56 ± 10
Man + MK	7	308 ± 12	0.16 ± 0.01	1.07 ± 0.04	15 ± 1	57 ± 8
Man + KT	7	312 ± 10	0.17 ± 0.01	1.08 ± 0.05	16 ± 2	60 ± 9
Man + DPCPX	7	278 ± 10	0.15 ± 0.01	1.05 ± 0.04	14 ± 2	65 ± 19
DPCPX	7	290 ± 17 *	0.16 ± 0.01	1.09 ± 0.04	15 ± 2	71 ± 10

Data are means ± SD. Con = control; Man = Mannitol; MK = MK2206 (Akt inhibitor); KT = KT5823 (PKG inhibitor); DPCPX = adenosine receptor 1 antagonist. * *p* < 0.05 vs. Man.

**Table 2:** Hemodynamic variables.

	Baseline	PC	Reperfusion	
			30	60
Heart Rate (bpm)				
Con	306 ± 26	281 ± 37	273 ± 43	255 ± 41
Man	296 ± 27	297 ± 22	278 ± 62	263 ± 21
Man + MK	306 ± 24	300 ± 34	308 ± 58	293 ± 69
Man + KT	318 ± 28	313 ± 28	273 ± 43	287 ± 33
Man + DPCPX	305 ± 37	311 ± 36	285 ± 68	248 ± 52
DPCPX	290 ± 34	298 ± 30	290 ± 18	246 ± 61
Left Ventricular Developed Pressure (mmHg)				
Con	139 ± 16	146 ± 14	22 ± 15 *	28 ± 10 *
Man	137 ± 16	135 ± 18	25 ± 6 *	34 ± 4 *
Man + MK	143 ± 16	141 ± 18	29 ± 14 *	34 ± 19 *
Man + KT	134 ± 6	137 ± 6	20 ± 10 *	31 ± 11 *
Man + DPCPX	141 ± 23	136 ± 23	18 ± 16 *	31 ± 11 *
DPCPX	136 ± 18	136 ± 17	23 ± 16 *	33 ± 17 *
Coronary flow (mL/min)				
Con	15 ± 2	15 ± 1	8 ± 1 *	7 ± 1 *
Man	18 ± 3	18 ± 4	10 ± 4 *	9 ± 3 *
Man + MK	16 ± 2	16 ± 2	10 ± 3 *	9 ± 2 *
Man + KT	14 ± 2	15 ± 1	8 ± 2 *	7 ± 2 *
Man + DPCPX	15 ± 1	15 ± 1	8 ± 1 *	8 ± 1 *
DPCPX	15 ± 3	15 ± 3	8 ± 3 *	7 ± 3 *

Data are means ± SD. PC = preconditioning; Con = control; Man = Mannitol; MK = MK2206 (Akt inhibitor); KT = KT5823 (PKG inhibitor); DPCPX = adenosine receptor 1 antagonist. * *p* < 0.05 versus baseline.

## Data Availability

The data presented in this study are available on request from the corresponding author.
